# High expression of neuroguidin increases the sensitivity of acute myeloid leukemia cells to chemotherapeutic drugs

**DOI:** 10.1186/s13045-015-0108-6

**Published:** 2015-02-19

**Authors:** Kejun Chen, Shuqing Lü, Hui Cheng, Gusheng Tang, Min Liu, Hong Zhou, Jianmin Wang

**Affiliations:** Department of Hematology, Changhai Hospital, Second Military Medical University, 168 Changhai Road, Shanghai, 200433 China

**Keywords:** Neuroguidin, Acute myeloid leukemia, Multidrug-resistant, Chemotherapeutic drug, mTOR

## Abstract

Neuroguidin (NGDN) is a eukaryotic translation initiation factor 4E binding protein. The purpose of this study was to clarify the function of NGDN and its possible mechanism of action in human myeloid leukemia cells. Proliferation inhibition and apoptosis in NGDN over-expressing myeloid multidrug-resistant leukemia cells (K562/A02-NGDN) was significantly higher than in control K562/A02 cells following treatment with vincristine, etoposide, and epirubicin, indicating that NGDN over-expression can increase the sensitivity of multidrug-resistant leukemia cells to chemotherapeutic drugs. Furthermore, NGDN knock-down in K562/A02 cells resulted in the activation of multiple tumor-related signaling pathways, especially the mammalian target of rapamycin (mTOR) pathway.

**Electronic supplementary material:**

The online version of this article (doi:10.1186/s13045-015-0108-6) contains supplementary material, which is available to authorized users.

## Findings

We previously reported the expression of some genes with unknown functions in myeloid leukemia cell lines and primary leukemia cells from clinical patients [1-3], one of which was homologous to neuroguidin (NGDN) [4]. Jung and colleagues confirmed that NGDN has a similar structure and function to eukaryotic translation initiation factor 4E (eIF4E) binding proteins [4], which are known to inhibit the cap-dependent protein translation as negative regulators of eIF4E and are involved in tumor cell proliferation, survival, and apoptosis [5-9]. Low expression and high phosphorylation of eIF4E binding protein 1 (4E-BP1) is associated with poor prognosis and tumor invasion [10]. High expression of 4E-BPs enhances tumor cell sensitivity to chemotherapeutic drugs and is associated with favorable clinical prognosis [11-13]. In this study, the effect of NGDN and its mechanism of action in human myeloid leukemia cells were investigated.

### Effects of NGDN over-expression on proliferation and apoptosis in multidrug-resistant leukemia cell line K562/A02

**Figure 1:**
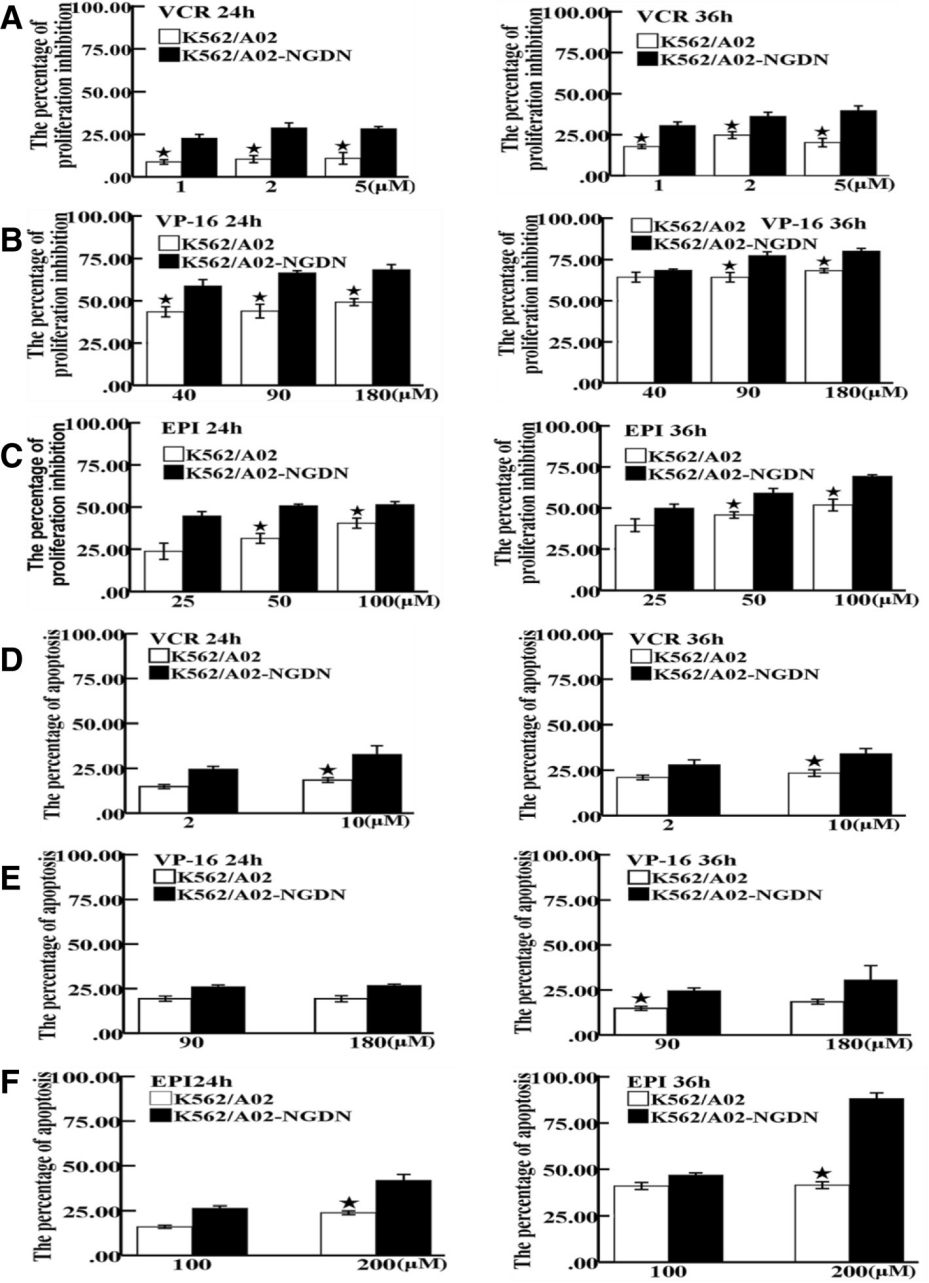
**Proliferation inhibition and apoptosis in NGDN over-expressing leukemia cells (K562/A02-NGDN) after chemotherapeutic drugs treatment.** The level of proliferation inhibition was examined using the CCK-8 assay following treatment with different concentrations of chemotherapeutic drugs for different lengths of time. The percentages of proliferation inhibition in K562/A02-NGDN and control K562/A02 cells after treatment with **(A)** vincristine (VCR), **(B)** etoposide (VP-16) and **(C)** epirubicin (EPI) are shown (mean ± SD, *n* = 3, star symbols: *P* < 0.05). The level of apoptosis was assessed using annexin V-FITC/allophycocyanin (APC ) staining by flow cytometry. The percentages of apoptosis in K562/A02-NGDN and control K562/A02 cells after treatment with **(D)** VCR, **(E)** VP-16, and **(F)** EPI are shown (mean ± SD, *n* = 3, star symbols: *P* < 0.05). NGDN: neuroguidin.

The human myeloid multidrug-resistant leukemia cell line K562/A02 was used to generate NGDN over-expressing leukemia cells (K562/A02-NGDN) by lentiviral transduction [see Additional files 1 and 2]. The proliferation of K562/A02-NGDN cells and control K562/A02 cells were assessed using the CCK-8 assay after treatment with different concentrations of vincristine (VCR), etoposide (VP-16), and epirubicin (EPI) for different lengths of time. Proliferation inhibition in K562/A02-NGDN cells was significantly higher than in control K562/A02 cells following treatment with each drug (*P* < 0.05) (Figure 1A,B,C). For example, after a 50-μM EPI treatment for 36 h, percent inhibition of K562/A02 and K562/A02-NGDN cell proliferation was 45.73% ± 1.93% and 59.15% ± 2.75%, respectively (*P* < 0.05) (Figure 1C). These results suggest that NGDN over-expression enhances the inhibitory effect of chemotherapeutic drugs on multidrug-resistant leukemia cell proliferation. Next, cell apoptosis was assessed using flow cytometry following treatment with different concentrations of VCR, VP-16, and EPI for different lengths of time. Apoptosis in K562/A02-NGDN cells was significantly higher than in K562/A02 cells (*P* < 0.05) (Figure 1D,E,F). For example, after a 200-μM EPI treatment for 24 h, the percentage of apoptosis detected in K562/A02 and K562/A02-NGDN cells was 23.85% ± 1.06% and 41.9% ± 3.25%, respectively (*P* < 0.05) (Figure 1F). These results suggest that NGDN over-expression can also enhance the apoptosis-inducing effect of chemotherapeutic drugs on multidrug-resistant leukemia cells. The effects of NGDN over-expression were also confirmed in human myeloid leukemia line K562 [see Additional files 1 and 3].

### Effect of NGDN knock-down in K562/A02 cells

To explore the mechanism of action of NGDN, a NGDN knock-down cell line (K562/A02-KD) was generated from K562/A02 cells by lentiviral transduction (transfected with small interfering RNA of NGDN), and a negative control was also generated with unrelated RNA (K562/A02-NC) [see Additional files 1 and 4]. The mRNA levels of core proteins of multiple pathways were detected by quantitative real-time reverse transcription PCR (qRT-PCR), including 4 housekeeping genes and 92 mammalian target of rapamycin (mTOR) pathway and mTOR-related pathway genes. The mTOR pathway is an important target in cancer therapy, as it is involved in the initiation of protein translation necessary for cell growth through the downstream effector 4E-BP1 and ribosomal protein S6 kinase [9,14]. qRT-PCR demonstrated a significant difference in the expression levels of 69 genes between K562/A02-KD and K562/A02-NC cells (*P* < 0.05). A number of genes were up-regulated in K562/A02-KD cells compared to K562/A02-NC cells (Table 1). For example, extracellular signaling genes were up-regulated including insulin-like growth factor 1(IGF1) and receptor of IGF1. The signal transduction pathway genes that were up-regulated in K562/A02-KD cells are involved in the mTOR pathway, the toll-like receptor signaling pathway, the mitogen-activated protein kinase pathway, the nuclear transcription factor kappa B pathway, and the Janus kinase/signal transducer and activator of the transcription signaling pathway. The up-regulated transcription factor genes include the nuclear factor of activated T cells, and the up-regulated cell invasion and metastasis-related genes include catenin and fibronectin 1. Oncogenes were also up-regulated in K562/A02-KD cells including MYC, Ras, and JUN. Some core genes of the mTOR pathway were significantly up-regulated in K562/A02-KD cells, including 3-phosphoinositide-dependent protein kinase-1, Akt, and mTOR. These results demonstrate that knock-down of the NGDN expression can activate many tumor-related signaling pathways (especially the mTOR pathway), which may promote tumor growth, angiogenesis and cell invasion, and inhibit apoptosis. The possible relationship between NGDN and the mTOR pathway was expounded in Additional files 1 and 5.

**Table 1:** **The main genes up-regulated in NGDN knock-down leukemia cells K562/A02-KD compared with negative control cells K562/A02-NC (n = 3,** ***P*** **< 0.05)**

**Category**	**Gene name**	**Relative mRNA expression level (K562/A02-KD/K562/A02-NC)**
Extracellular signal gene	IGF1	1.7
	IGF1R	1.7
Signal transduction pathway gene	mTOR	7.9
	Akt	13.7
	PDPK1	3.0
	TSC1	1.9
	PKC	1.7
	IL1R1	2.6
	TOLLIP	1.8
	MAPK3	1.5
	NF-κB2	2.4
	STAT2	2.1
Transcription factor gene	NFAT	2.9
	ELK1	2.5
	E2F1	1.5
	HSF1	1.6
Cell invasion and metastasis-related gene	CTNN	2.9
	FN1	2.1
	ITGAM	1.5
	VIM	1.7
Oncogene	MYC	1.5
	Ras	1.5
	JUN	3.6

*IGF1* insulin-like growth factor 1, *IGF1R* receptor of insulin-like growth factor 1, *mTOR* mammalian target of rapamycin, *Akt* protein kinase B, *PDPK1* 3-phosphoinositide-dependent protein kinase-1, *TSC1* tuberous sclerosis complex 1, *PKC* protein kinase C, *IL1R1* receptor 1 of interleukin 1, *TOLLIP* toll interacting protein, *MAPK3* mitogen-activated protein kinase 3, *NF-κB2* Nuclear transcription factor-kappa B 2, *STAT2* signal transducer and activator of transcription 2, *NFAT* nuclear factor of activated T cells, *ELK1* ETS-like gene 1, *E2F1* E2F transcription factor 1, *HSF1* heat shock factor 1, *CTNN* catenin, *FN1* fibronectin 1, *ITGAM* integrin alpha-M, *VIM* vimentin.

Overall, the results of this study *in vitro* confirmed that NGDN over-expression can increase the sensitivity of human myeloid multidrug-resistant leukemia cells to chemotherapeutic drugs, indicating that the high expression of NGDN may be a favorable prognostic factor for patients with acute myeloid leukemia [see Additional file 1]. The specific mechanism of action of NGDN in leukemia cells requires further study.

## Additional files

Additional file 1:
**Supplementary data.** The details of neuroguidin (NGDN) over-expressing human myeloid leukemia cells and NGDN knock-down leukemia cells generated by lentivirus transduction were shown. The study in vitro confirmed that NGDN over-expression can increase the sensitivity of human myeloid leukemia cells K562 to chemotherapeutic drugs. The possible relationship between NGDN and mammalian target of the rapamycin (mTOR) pathway was expounded. We inferred that NGDN can be regulated by the mTOR pathway and also regulate the mTOR pathway in a way of negative feedback. The results of the study on acute myeloid leukemia (AML) patients suggest that high NGDN mRNA expression level may be relative to the low bone marrow blast cell proportion and less inducing chemotherapy courses to obtain complete remission in AML patients. Additional file 2: Figure S1. The NGDN over-expressing human myeloid leukemia cells K562-NGDN and K562/A02-NGDN generated by lentivirus transduction. K562-NGDN and K562/A02-NGDN: NGDN over-expressing leukemia cells generated from human myeloid leukemia cell line K562 and its multidrug-resistant subline K562/A02. K562-CON: Negative control K562 cells transfected with empty vector. The green fluorescent protein (GFP) expressed in K562-NGDN, K562-CON, and K562/A02-NGDN cells was observed by fluorescence microscope **(A)** and flow cytometry **(B)**. The mRNA expression levels of the NGDN gene detected by real-time fluorescent quantitative reverse transcription-polymerase chain reaction in K562-NGDN, K562-CON, and K562 cells were shown in **(C)**; the mRNA expression levels of NGDN in K562/A02-NGDN and K562/A02 cells were shown in **(D)**. Additional file 3: Figure S2. Proliferation inhibition and apoptosis in NGDN over-expressing leukemia cells (K562-NGDN) after chemotherapeutic drug treatment. K562-CON: negative control cells transfected with an empty vector. Proliferation inhibition was examined using the CCK-8 method after treatment with different concentrations of chemotherapeutic drugs for different lengths of time. The percentages of proliferation inhibition in K562-NGDN and control cells after treatment with **(A)** vincristine (VCR),** (B)** etoposide (VP-16), **(C)** daunorubicin (DNR), and** (D)** epirubicin (EPI) are shown (mean ± SD, n = 3, star symbols: P < 0.05). The level of apoptosis was assessed using annexin V-FITC/APC staining by flow cytometry. The percentages of apoptosis in K562-NGDN and control cells after treatment with **(E)** VCR, **(F)** VP-16,** (G)** (DNR), and **(H)** EPI are shown (mean ± SD, n = 3, star symbols: P < 0.05). Additional file 4: Figure S3. The NGDN knock-down cells K562/A02-KD and negative control cells K562/A02-NC. The green fluorescent protein (GFP) expressed in K562/A02-KD **(A)** and K562/A02-NC **(B)** cells was observed by fluorescence microscope. **(C)** The mRNA expression levels of NGDN in K562/A02-KD and K562/A02–NC cells were detected by fluorescent quantitative reverse transcription-polymerase chain reaction. Additional file 5: Figure S4. The possible relationship between NGDN and mTOR pathway. eIF4E: eukaryotic translation initiation factor 4E; 4EBP1: eIF4E binding protein 1; mTOR: mammalian target of rapamycin; IGF-1: insulin-like growth factors 1; IRS-1: insulin receptor substrate 1; PDPK1: 3-phosphoinositide -dependent protein kinase-1; PI3K: phosphatidylinositol 3-kinase; PKB/Akt: protein kinase B; RPS6K: ribosomal protein S6 kinase. mTORC1 phosphorylated the P70S6K and 4EBP1 when the insulin activated PI3K and mTOR in turn. Then, P70S6K promoted the degradation of IRS-1 by way of negative feedback and eventually inhibited the IRS-1-mediated activation of the mTOR pathway. NGDN may be regulated by the mTOR pathway and also regulate the mTOR pathway in a way of negative feedback similar to P70S6K/4EBP1. The red font represents the possible NGND-regulated pathways.
