# Supplementary material for: High expression of neuroguidin increases the sensitivity of acute myeloid leukemia cells to chemotherapeutic drugs
Source: J Hematol Oncol. 2015 Feb 19;8:11. doi: 10.1186/s13045-015-0108-6 (PMC4340276; doi:10.1186/s13045-015-0108-6)
Supplement: Additional file 1: — Supplementary data. The details of neuroguidin (NGDN) over-expressing human myeloid leukemia cells and NGDN knock-down leukemia cells generated by lentivirus transduction were shown. The study in vitro confirmed that NGDN over-expression can increase the sensitivity of human myeloid leukemia cells K562 to chemotherapeutic drugs. The possible relationship between NGDN and mammalian target of the rapamycin (mTOR) pathway was expounded. We inferred that NGDN can be regulated by the mTOR pathway and also regulate the mTOR pathway in a way of negative feedback. The results of the study on acute myeloid leukemia (AML) patients suggest that high NGDN mRNA expression level may be relative to the low bone marrow blast cell proportion and less inducing chemotherapy courses to obtain complete remission in AML patients. [file 13045_2015_108_MOESM1_ESM.docx]

**Supplementary** **data**

**NGDN over-expressing human myeloid leukemia cells K562-NGDN and K562/A02-NGDN were generated by lentivirus transduction**

NGDN over-expressing leukemia cells K562-NGDN and K562/A02-NGDN were generated by lentivirus transduction from human myeloid leukemia cell line K562 and its multidrug-resistant subline K562/A02. Negative control cell K562-CON was also generated (transfected with empty vector). The green fluorescent protein (GFP) expressed in K562-NGDN and K562/A02-NGDN cells was detected by fluorescence microscope (Fig. S1 A) and flow cytometry (Fig. S1 B). The mRNA expression level of NGDN gene in K562-NGDN cells was 8.2 times compared with K562 cells (Figure S1 C) and the expression level in K562/A02-NGDN cells was 11.3 times compared with K562/A02 cells (Fig. S1 D).

**Effects of NGDN over-expression on proliferation and apoptosis in leukemia cell line K562**

Proliferation of K562-NGDN cells and control K562 cells was assessed using the CCK-8 assay after chemotherapeutic drugs treatment. After treatment with different concentrations of VCR, VP-16, DNR and EPI for different lengths of time, proliferation inhibition in K562-NGDN cells was significantly higher than in K562 and K562-CON cells following treatment with each drug (*P* < 0.05) (Fig. S2 A-D). For example, after 20μM VP-16 treatment for 36h, percent inhibition of K562, K562-CON and K562-NGDN cell proliferation was 43.06% ± 2.67%, 46.50% ± 2.89% and 65.10% ± 3.65% respectively (K562-NGDN *vs* K562, *P* < 0.05; K562-NGDN *vs* K562-CON, *P* < 0.05) (Fig. S2 B).

Next, cell apoptosis was assessed using flow cytometry following chemotherapeutic drugs treatment. After treatment with different concentrations of VCR, VP-16, DNR and EPI for different lengths of time, apoptosis in K562-NGDN cells was significantly higher than in K562 and K562-CON cells (Fig. S2 E-H). For example, after 4μM DNR treatment for 48h, the percentage of apoptosis in K562, K562-CON and K562-NGDN cells was 40.03% ± 3.52%, 41.53% ± 3.25% and 55.84% ± 2.61% respectively (K562-NGDN *vs* K562, *P* < 0.05; K562-NGDN *vs* K562-CON, *P* < 0.05) (Fig. S2 G).

These results confirmed that NGDN over-expression can increase the sensitivity of human myeloid leukemia cell K562 to chemotherapeutic drugs in vitro.

**NGDN knock-down cell line (K562/A02-KD) was generated using small RNA interference technology.**

NGDN knock-down cells (K562/A02-KD) and negative control (K562/A02-NC) were generated from K562/A02 cells using small RNA interference technology. The GFP expressed in K562/A02-KD (Fig. S3 A) and K562/A02-NC (Fig. S3 B) cells was observed by fluorescence microscope. The relative mRNA expression level of NGDN gene in K562/A02-KD cells was 29.1% ± 0.5% compared with K562/A02-NC cells. In other word, the efficiency of NGDN knock-down was 70.9% ± 0.5% (Fig. S3 C).

**The possible relationship between NGDN and mTOR pathway**

It is notable that NGDN knock-down caused remarkable up-regulated expression of some mTOR pathway genes as shown in Table 1. We inferred that NGDN can be regulated by mTOR pathway and also regulate mTOR pathway in a way of negative feedback similar to 4EBP1/ ribosomal protein S6 kinase (P70S6K) (Fig. S4). The reasons are as follows. Firstly, mRNA levels of some core genes of the mTOR pathway were increased remarkably when NGDN was inhibited. Secondly, the function of NGDN is similar to 4EBP1, which was known to be regulated by PI3K-Akt-mTOR pathway [5]. Thirdly, previous research suggested that mTORC1 phosphorylated the P70S6K/4EBP1 when insulin activated PI3K and mTOR in turn. Then P70S6K promoted the degradation of insulin receptor substrate-1 (IRS-1) by way of negative feedback and eventually inhibited IRS-1-mediated activation of mTOR pathway [14].

The relationship between NGDN and mTOR pathway will be further studied in NGDN over-expression leukemia cell lines K562-NGDN and K562/A02-NGDN at the mRNA and protein levels.

**The relationship between** **NGDN mRNA expression levels and clinical characteristics of acute myeloid leukemia (AML) patients**

The NGDN mRNA expression levels in bone marrow mononuclear cells from 59 de novo AML patients and 15 [healthy volunteers](app:ds:healthy%20volunteers) were detected by qRT-PCR. The average expression level of AML patients was 4.3（median 0.04-67.36）relative to housekeeping gene GAPDH while the level of [healthy volunteers](app:ds:healthy%20volunteers) was 1.7（median 0.48-3.82）. According to the mRNA expression level, 59 AML patients were divided into NGDN low-expression group (< 1.7, n=25) and NGDN high-expression group (≥ 1.7, n=34). The results showed that there were no significant differences between these two groups in age, gender, fever, anemia, bleeding, invasion outside the bone marrow, white blood cell count, platelet count, FAB classification, molecular and immunological phenotype (*P* > 0.05). The bone marrow blast cell proportions of 58.8% (20/34) patients were < 70% in NGDN high-expression group, but only 28% (7/25) patients < 70% in NGDN [low-expression](app:ds:high%20expression) group (*P* = 0.019).The proportions of peripheral blood blast cells of 16 (47%) patients were < 30% in NGDN high-expression group，but only 6 (24%) patients were < 30% in NGDN [low-expression](app:ds:high%20expression) group (P = 0.070). The 67.6% (23/34) patients only need one course of standard induction chemotherapy to achieve complete remission (CR) in NGDN high-expression group，but only 44%（11/25）patients achieved CR after one course of standard chemotherapy in NGDN [low-expression](app:ds:high%20expression) group (P=0.069)（Table S1）. These results suggest that high NGDN mRNA expression level may be relative to the low bone marrow blast cell proportion and less [inducing chemotherapy](app:ds:inducing%20chemotherapy) courses to obtain CR in AML patients. In other words, NGDN high expression may inhibit the proliferation of human leukemia cells and increase the sensitivity of leukemia cells to chemotherapeutic drugs in vivo, indicating that high expression of NGDN may be a favorable prognostic factor for AML patients. To confirm the clinical prognostic significance of NGDN for AML patients, further larger scale clinical research is required.

**Figure legends**

**Figure S1. The NGDN over-expressing human myeloid leukemia cells K562-NGDN and K562/A02-NGDN generated by lentivirus transduction**

**K562-NGDN and K562/A02-NGDN**: NGDN over-expressing leukemia cells generated by lentivirus transduction from human myeloid leukemia cell line K562 and its multidrug-resistant subline K562/A02. **K562-CON**: Negative control K562 cells transfected with empty vector. The green fluorescent protein (GFP) expressed in K562-NGDN, K562-CON and K562/A02-NGDN cells was observed by fluorescence microscope (**A**) and flow cytometry (**B**). The mRNA expression levels of NGDN gene detected by real-time fluorescent quantitative reverse transcription-polymerase chain reaction in K562-NGDN, K562-CON and K562 cells were shown in (**C**); the mRNA expression levels of NGDN in K562/A02-NGDN and K562/A02 cells were shown in (**D**).

**Figure S2. Proliferation inhibition and apoptosis in NGDN over-expressing leukemia cells (K562-NGDN) after chemotherapeutic drugs treatment.**

**K562-NGDN**: NGDN over-expressing leukemia cells constructed by lentivirus transduction from human myeloid leukemia cell line K562. **K562-CON**: negative control cells transfected with empty vector. Proliferation inhibition was examined using CCK-8 method after treatment with different concentrations of chemotherapeutic drugs for different lengths of time. The percentages of proliferation inhibition in K562-NGDN and control cells after treatment with (**A**) vincristine (VCR), (**B**) etoposide (VP-16), (**C**) daunorubicin (DNR) and (**D**) epirubicin (EPI) are shown (mean ± SD, n = 3, ^★^*p* < 0.05). The level of apoptosis was assessed using annexinV-FITC/APC staining by flow cytometry. The percentages of apoptosis in K562-NGDN and control cells after treatment with (**E**) VCR, (**F**) VP-16, (**G**) (DNR) and (**H**) EPI are shown (mean ± SD, n = 3, ^★^*p* < 0.05).

**Figure S3. The NGDN knock-down cells K562/A02-KD and negative control cells K562/A02-NC.**

**K562/A02-KD**: NGDN knock-down leukemia cells (transfected with small interfering RNA of NGDN by lentivirus vector from multidrug-resistant leukemia line K562/A02). **K562/A02-NC**: negative control (transfected with unrelated RNA). The green fluorescent protein (GFP) expressed in K562/A02-KD (**A**) and K562/A02-NC (**B**) cells was observed by fluorescence microscope. (**C**)The mRNA expression levels of NGDN in K562/A02-KD and K562/A02–NC cells were detected by fluorescent quantitative reverse transcription-polymerase chain reaction.

**Figure S4. The possible relationship between NGDN and mTOR pathway**

eIF4E: eukaryotic translation initiation factor 4E; 4EBP1: eIF4E binding protein 1; mTOR: mammalian target of rapamycin; IGF-1: insulin-like growth factors 1; IRS-1: insulin receptor substrate 1; PDPK1: 3-phosphoinositide -dependent protein kinase-1; PI3K: phosphatidylinositol 3-kinase; PKB/Akt: protein kinase B; RPS6K: ribosomal protein S6 kinase.

mTORC1 phosphorylated the P70S6K and 4EBP1 when insulin activated PI3K and mTOR in turn. Then P70S6K promoted the degradation of IRS-1 by way of negative feedback and eventually inhibited IRS-1-mediated activation of mTOR pathway. NGDN may be regulated by mTOR pathway and also regulate mTOR pathway in a way of negative feedback similar to P70S6K/4EBP1. The red font represents the possible NGND-regulated pathways.

**Table S1. The clinical characteristics of NGDN low-expression and high-expression patients with de novo acute myeloid leukemia (n=59)**

| **Characteristics** | **Category** | **NGDN low-expression** | **NGDN high-expression** | ***P* value** |
| --- | --- | --- | --- | --- |
| Gender | Male  Female | 11  14 | 22  12 | 0.113 |
| Age（y） | <60  >60 | 22  3 | 28  6 | 0.72 |
| Fever | Yes  No | 14  11 | 16  18 | 0.497 |
| Anemia | Yes  No | 9  16 | 11  23 | 0.77 |
| Bleeding | Yes  No | 8  17 | 10  24 | 0.831 |
| Invasion outside bone marrow | Yes  No | 17  8 | 27  7 | 0.32 |
| White blood cell count (×10^9^/L) | <30  ≥30 | 15  10 | 21  13 | 0.891 |
| Genetic risk group | Favorable  Intermediate  Unfavorable | 4  17  4 | 7  16  11 | 0.334 |
| **Bone marrow blast cells** | **<70%**  **≥70%** | **7**  **18** | **20**  **14** | **0.019** |
| **Peripheral blood blast cells** | **<30%**  **≥30%** | **6**  **19** | **16**  **18** | **0.07** |
| **The number of courses to achieve complete remission** | **1**  **>1** | **11**  **14** | **23**  **11** | **0.069** |
