# Supplementary figures and images for: High expression of neuroguidin increases the sensitivity of acute myeloid leukemia cells to chemotherapeutic drugs
Source: J Hematol Oncol. 2015 Feb 19;8:11. doi: 10.1186/s13045-015-0108-6 (PMC4340276; doi:10.1186/s13045-015-0108-6)

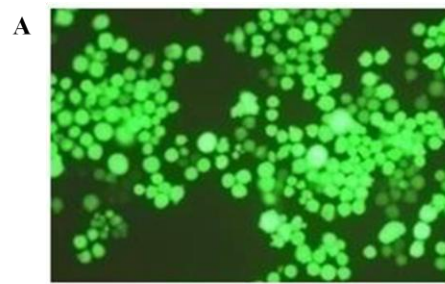

K562-NGDN (200×10)

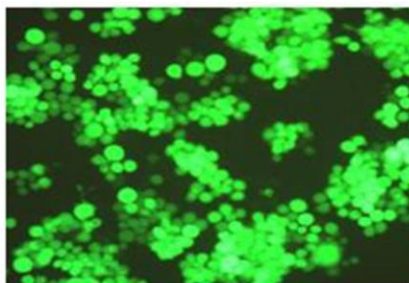

K562/A02-NGDN (200×10)

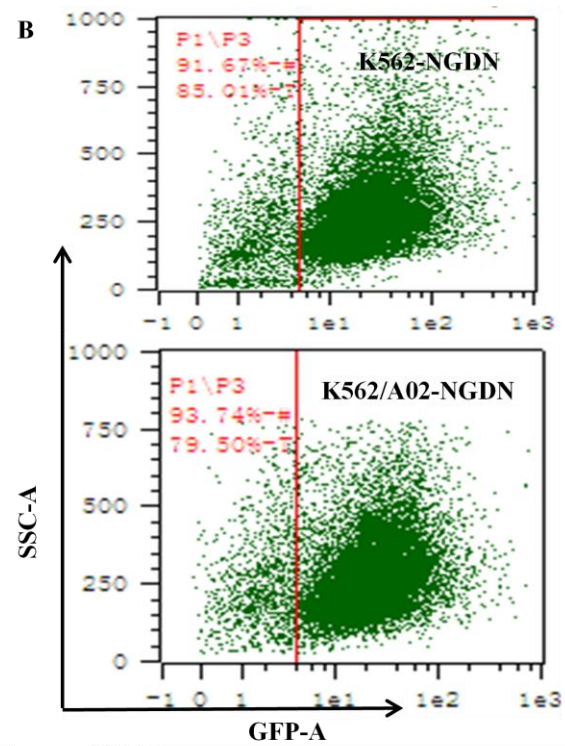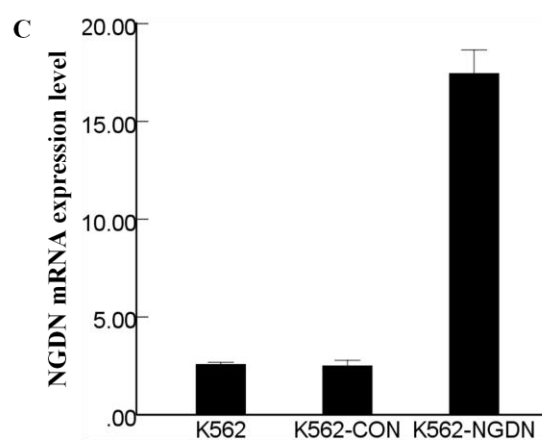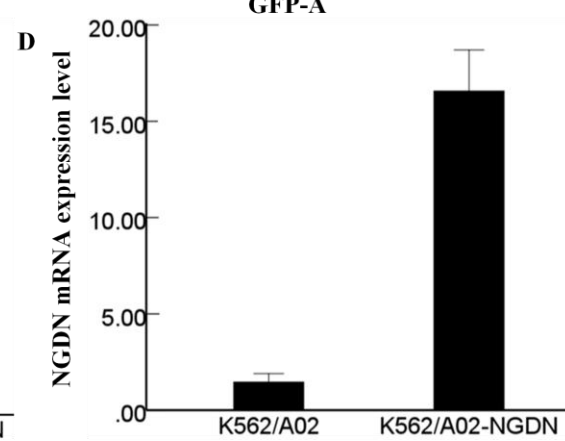

Supplement: Additional file 2: Figure S1. — The NGDN over-expressing human myeloid leukemia cells K562-NGDN and K562/A02-NGDN generated by lentivirus transduction. K562-NGDN and K562/A02-NGDN: NGDN over-expressing leukemia cells generated from human myeloid leukemia cell line K562 and its multidrug-resistant subline K562/A02. K562-CON: Negative control K562 cells transfected with empty vector. The green fluorescent protein (GFP) expressed in K562-NGDN, K562-CON, and K562/A02-NGDN cells was observed by fluorescence microscope (A) and flow cytometry (B). The mRNA expression levels of the NGDN gene detected by real-time fluorescent quantitative reverse transcription-polymerase chain reaction in K562-NGDN, K562-CON, and K562 cells were shown in (C); the mRNA expression levels of NGDN in K562/A02-NGDN and K562/A02 cells were shown in (D). [file 13045_2015_108_MOESM2_ESM.pdf]

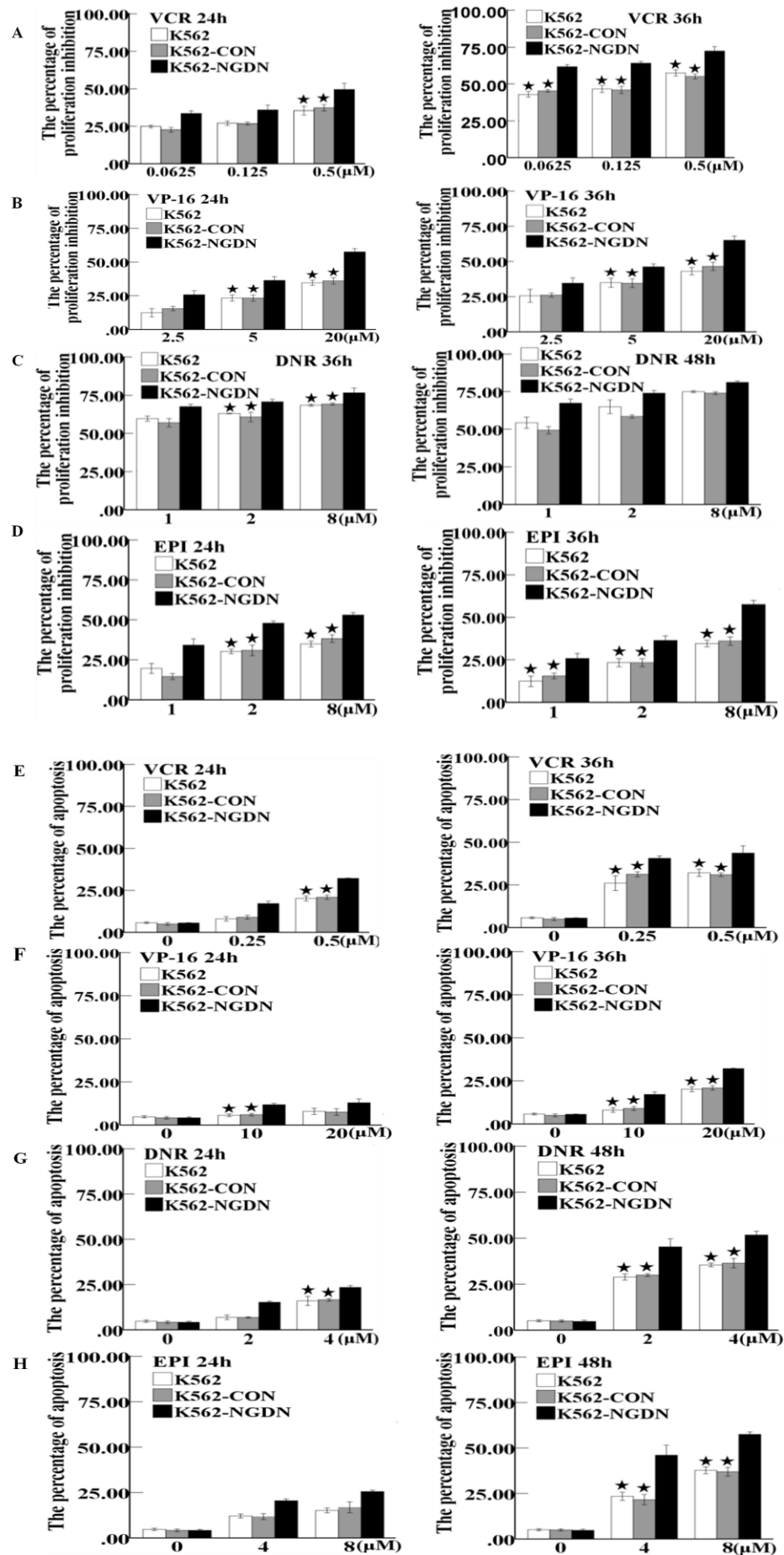

Supplement: Additional file 3: Figure S2. — Proliferation inhibition and apoptosis in NGDN over-expressing leukemia cells (K562-NGDN) after chemotherapeutic drug treatment. K562-CON: negative control cells transfected with an empty vector. Proliferation inhibition was examined using the CCK-8 method after treatment with different concentrations of chemotherapeutic drugs for different lengths of time. The percentages of proliferation inhibition in K562-NGDN and control cells after treatment with (A) vincristine (VCR), (B) etoposide (VP-16), (C) daunorubicin (DNR), and (D) epirubicin (EPI) are shown (mean ± SD, n = 3, star symbols: P < 0.05). The level of apoptosis was assessed using annexin V-FITC/APC staining by flow cytometry. The percentages of apoptosis in K562-NGDN and control cells after treatment with (E) VCR, (F) VP-16, (G) (DNR), and (H) EPI are shown (mean ± SD, n = 3, star symbols: P < 0.05). [file 13045_2015_108_MOESM3_ESM.pdf]

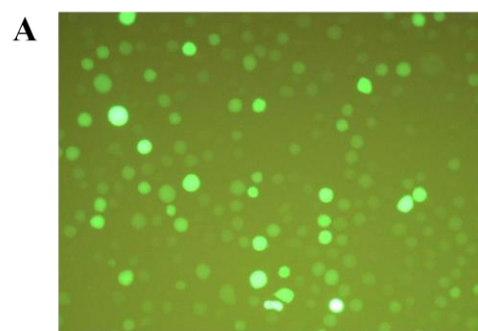

**K562/A02-KD (200×10)**

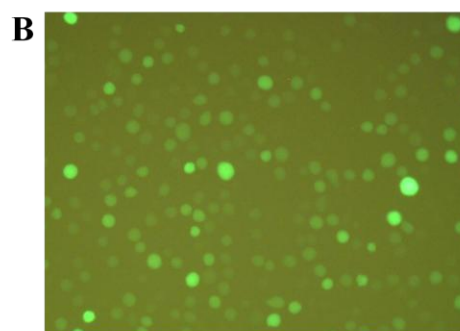

**K562/A02-NC (200×10)**

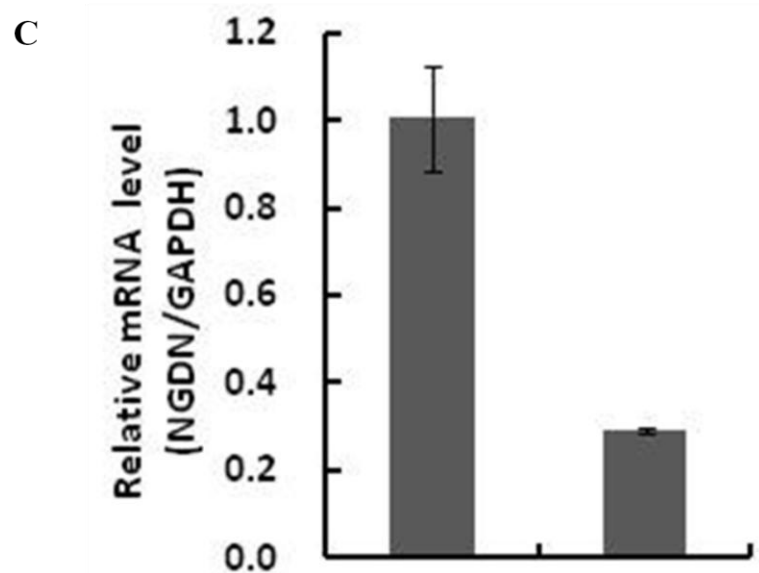

Supplement: Additional file 4: Figure S3. — The NGDN knock-down cells K562/A02-KD and negative control cells K562/A02-NC. The green fluorescent protein (GFP) expressed in K562/A02-KD (A) and K562/A02-NC (B) cells was observed by fluorescence microscope. (C) The mRNA expression levels of NGDN in K562/A02-KD and K562/A02–NC cells were detected by fluorescent quantitative reverse transcription-polymerase chain reaction. [file 13045_2015_108_MOESM4_ESM.pdf]

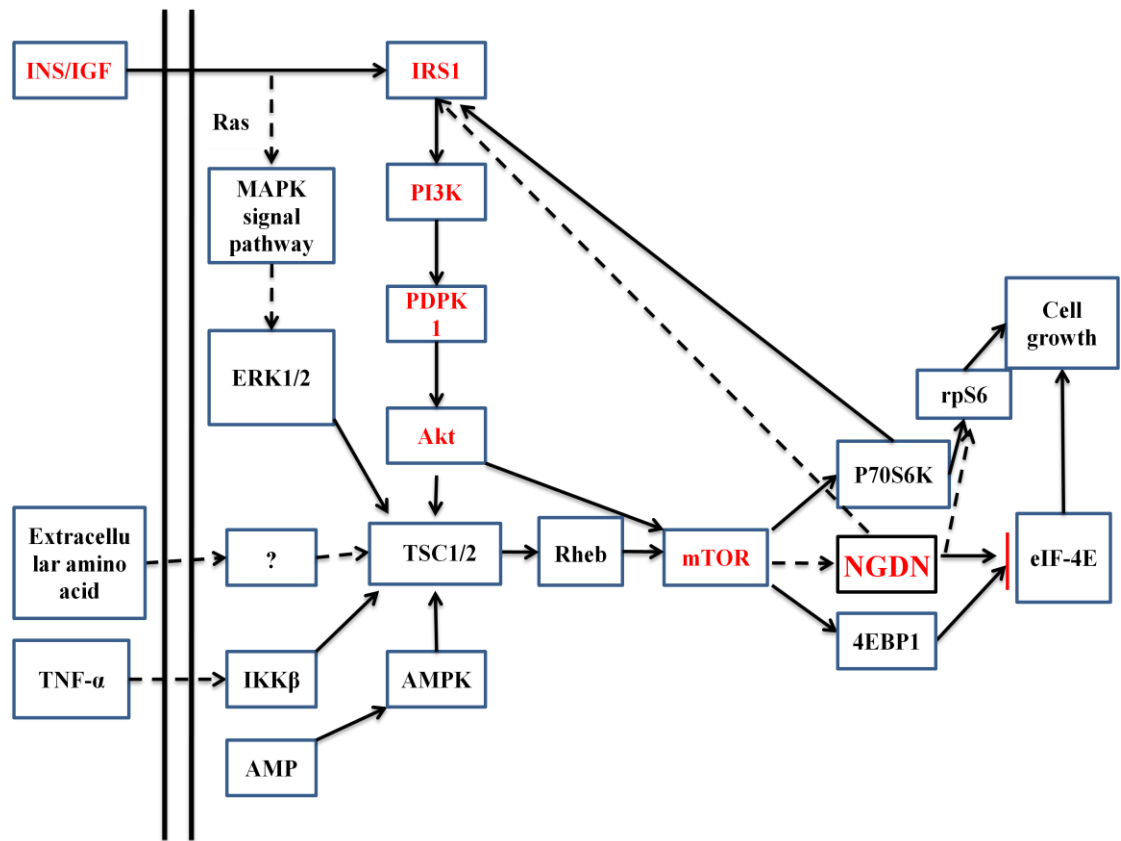

Supplement: Additional file 5: Figure S4. — The possible relationship between NGDN and mTOR pathway. eIF4E: eukaryotic translation initiation factor 4E; 4EBP1: eIF4E binding protein 1; mTOR: mammalian target of rapamycin; IGF-1: insulin-like growth factors 1; IRS-1: insulin receptor substrate 1; PDPK1: 3-phosphoinositide -dependent protein kinase-1; PI3K: phosphatidylinositol 3-kinase; PKB/Akt: protein kinase B; RPS6K: ribosomal protein S6 kinase. mTORC1 phosphorylated the P70S6K and 4EBP1 when the insulin activated PI3K and mTOR in turn. Then, P70S6K promoted the degradation of IRS-1 by way of negative feedback and eventually inhibited the IRS-1-mediated activation of the mTOR pathway. NGDN may be regulated by the mTOR pathway and also regulate the mTOR pathway in a way of negative feedback similar to P70S6K/4EBP1. The red font represents the possible NGND-regulated pathways. [file 13045_2015_108_MOESM5_ESM.pdf]
